# Case report: PCA-2-associated encephalitis with different clinical phenotypes: a two-case series and literature review

**DOI:** 10.3389/fimmu.2024.1431585

**Published:** 2024-07-12

**Authors:** Xiaona Li, Yue Lang, Di Ma, Jing Bai, Pingping Shen, Xinyu Wang, Li Cui

**Affiliations:** Department of Neurology, The First Hospital of Jilin University, Changchun, China

**Keywords:** PCA-2, MAP1B, limbic encephalitis, autoimmune cerebellitis, paraneoplastic neurologic syndrome, PET-CT

## Abstract

Purkinje cell cytoplasmic antibody type 2 (PCA-2), identified in 2000, targets the widely distributed microtubule-associated protein 1B in the central and peripheral nervous systems, leading to diverse clinical phenotypes of neurological disorders. We report two cases of PCA-2-associated encephalitis, each presenting with distinct onset forms and clinical manifestations, thereby illustrating the phenotypic variability of PCA-2-related diseases. The first patient was diagnosed with PCA-2-associated autoimmune cerebellitis and undifferentiated small cell carcinoma with metastasis in mediastinal lymph nodes of unknown primary origin. The second patient was diagnosed with PCA-2-associated limbic encephalitis. Our findings underscore the superior sensitivity of positron emission tomography-computed tomography over brain magnetic resonance imaging in the early detection of PCA-2-associated encephalitis. Given the high risk of relapse and suboptimal response to traditional immunotherapy in PCA-2-related neurological disorders, this study highlights the need for a deeper understanding of their pathogenesis to develop more effective treatments to control symptoms and improve patient prognosis.

## 1 Introduction

Purkinje cell cytoplasmic antibody type 2 (PCA-2), a newly discovered paraneoplastic antibody, was first reported by Vernino et al. in 2000 (1). Its target antigen, the microtubule-associated protein 1B (MAP1B) (2), is widely distributed in the central and peripheral nervous systems, leading to various clinical disease phenotypes. The most common of these include peripheral neuropathy, cerebellar ataxia, and encephalopathy/cognitive decline (1–3). Paraneoplastic neurologic syndrome (PNS) is an indirect effect of cancer on the nervous system that is not caused by the direct invasion or secondary effects of the tumor and its metastases. It is usually associated with the presence of specific serum antibodies and can involve any part of the nervous system. The current diagnostic criteria for PNS classify PCA-2 as a high-risk antibody (>70% associated with cancer). The most common malignancies associated with PCA-2 are small-cell lung cancer, non-small-cell lung cancer, and breast cancer (4). Despite PCA-2 being the seventh most common intracellular antigen antibody, reports on the phenotypes of nervous system diseases caused by PCA-2 are relatively scarce, likely owing to its low detection rate of only 0.024% (2). Our center recently admitted two patients diagnosed with PCA-2-associated encephalitis who presented with completely different onset forms and clinical manifestations. Here, we report these two cases and retrospectively analyze PCA-2-related neurological diseases.

## 2 Case description

### 2.1 Case 1

A 57-year-old male patient, a smoker for over 20 years, presented with an unsteady gait lasting 1 month and dizziness worsening over the past 5 days. Gait instability was manifested by his inability to walk in a straight line, whereas dizziness occurred independently of body position, occasionally accompanied by blurred vision. There were no reports of nausea, vomiting, tinnitus, hearing loss, recent unconscious episodes, limb convulsions, or significant changes in urinary or fecal continence or weight. Neurological examination revealed responsiveness, dysarthria (poetic-like language), pronounced horizontal nystagmus in both eyes, and unsteady bilateral finger-to-nose and heel-knee-tibia tests. The Romberg sign was positive with both eyes open and closed; other findings were normal. Laboratory tests indicated an elevated carcinoembryonic antigen level at 10.44 ng/mL (normal value: <5.0 ng/mL**).**

**Figure 1:**
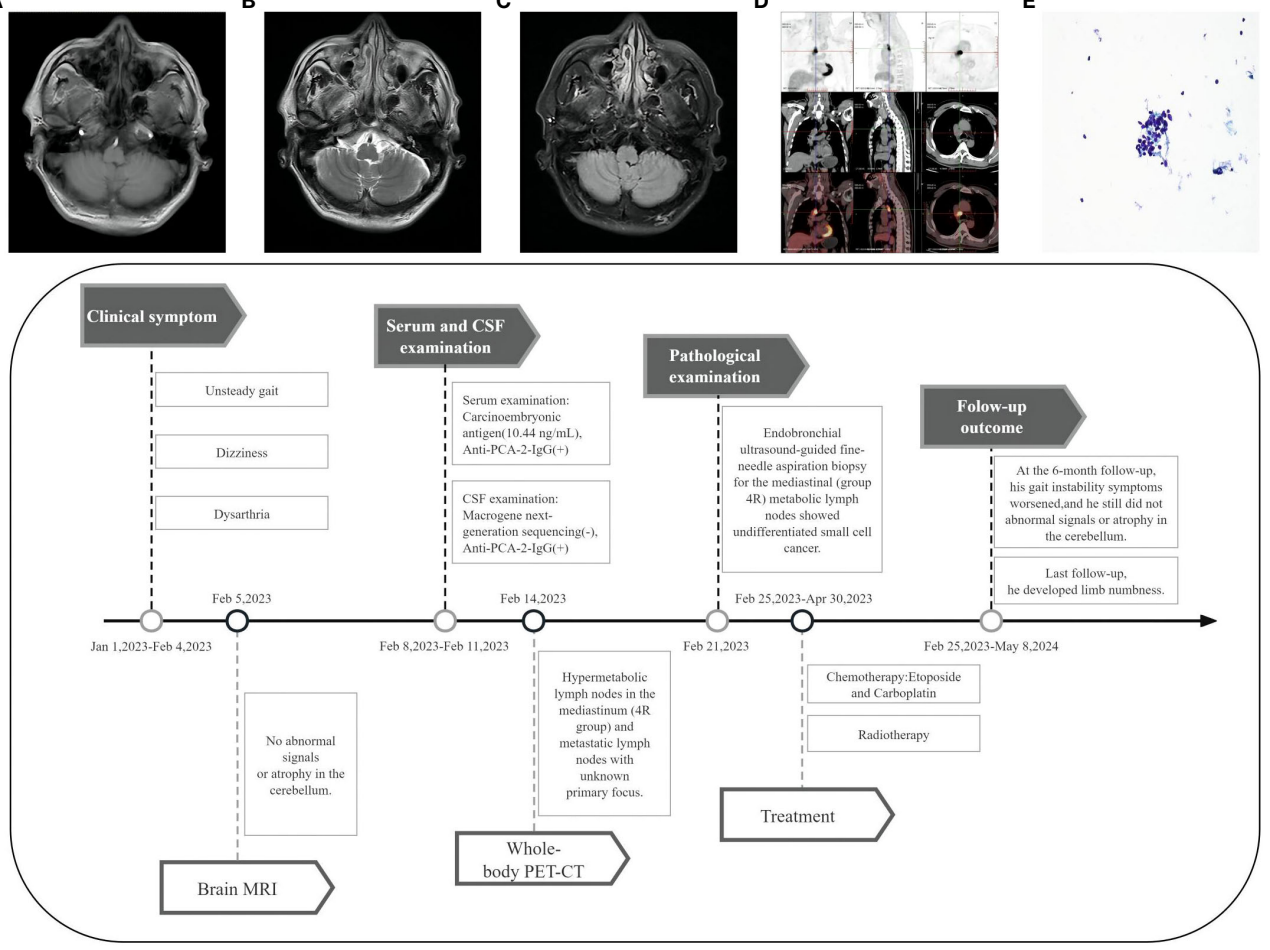
Imaging and clinical course of Case 1. **(A-C)** Brain MRI showed no abnormal signals or atrophy in the cerebellum. **(D)** PET-CT showed hypermetabolic lymph nodes in the mediastinum (4R group), with the largest measuring 3.2 cm × 2.1 cm. **(E)** Fine-needle aspiration cytology of the mediastinal lymph node (Papanicolaou stain [× 400 magnification]) showed undifferentiated small cell carcinoma cells. **(F)** Timeline of the clinical course in Case 1.

**Figure 2:**
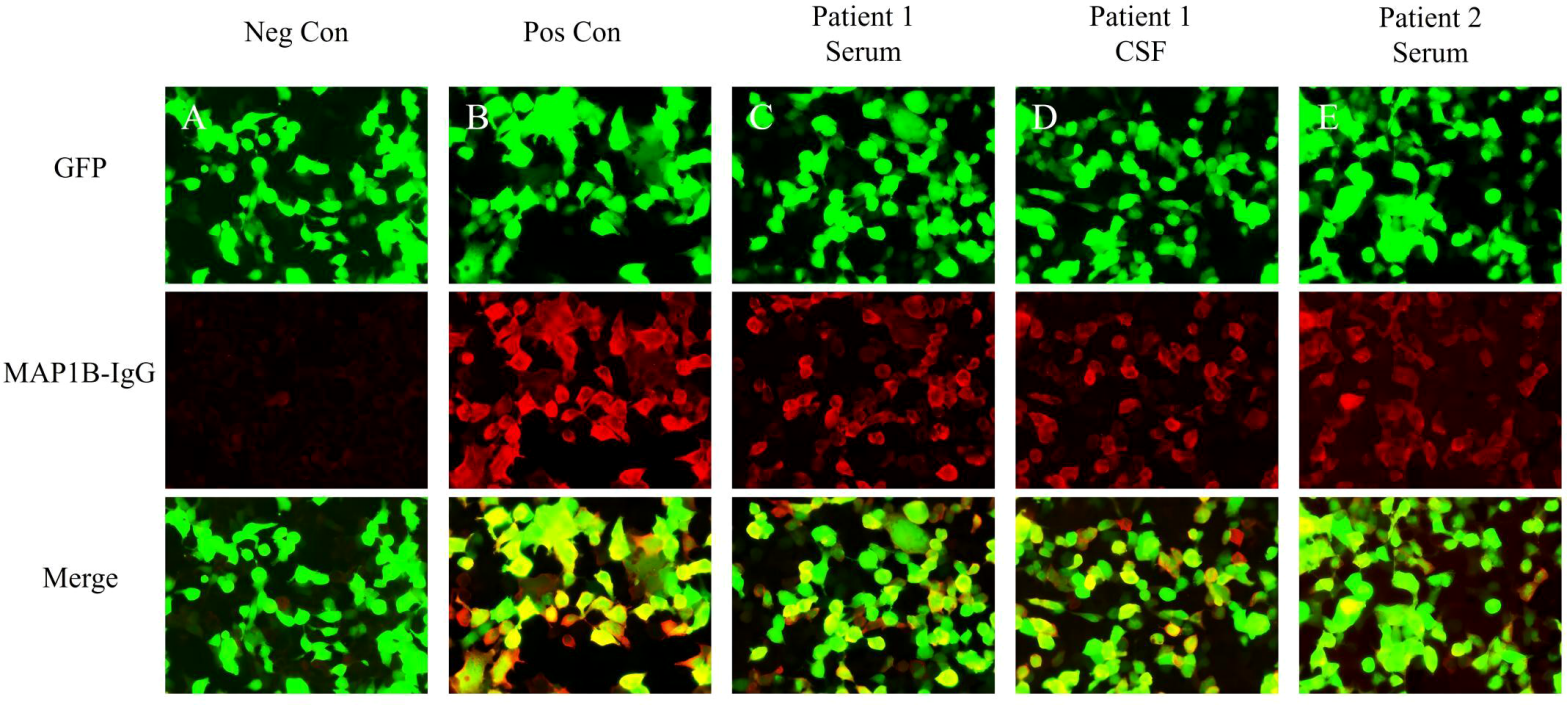
Test results of serum and CSF using indirect immunofluorescence assays. **(A)** Negative controls without MAP1B antibodies in the serum. **(B)** Positive controls from a patient with classical anti-MAP1B. **(C)** Serum MAP1B-IgG test results (1:30) of Patient 1. **(D)** CSF MAP1B-IgG test results (1:30) of Patient 1. **(E)** Serum MAP1B-IgG test results (1:30) of Patient 2. GFP, green fluorescent protein; Neg Con, negative control; Pos Con, positive control.

Brain magnetic resonance imaging (MRI) showed no abnormal signals or atrophy in the cerebellum (**Figures 1A–C**). Cerebrospinal fluid (CSF) examination by lumbar puncture showed elevated protein levels (0.63 g/L [reference range: 0.15–0.45 g/L]), white blood cell count (40×10^6/L [reference range: 0–8×10^6/L]), and immunoglobulin G (36.63 mg/L [reference range: 0–34 mg/L]). Macrogene next-generation sequencing of the CSF was negative, but the cell-based assay revealed positivity for anti-PCA-2 (MAP1B) in serum and CSF (both titers 1:30, **Figures 2A–D**). Additionally, anti-mGluR1, mGluR2, mGluR8, KLHL11, Homer3, ARHGAP26, ATP1A3, CARP VII, GluR δ2, CASPR2, Yo, Hu, Ri, CV2, Ma1, Ma2, amphiphysin, SOX1, Tr, Zic4, recoverin, titin, PKCγ, and GAD65 were tested negative. The positive anti-PCA-2 strongly indicated a lung tumor, confirmed by both plain and contrast-enhanced lung computed tomography (CT). Nodules (~1.0 cm) of undetermined nature were seen in the left upper lobe of the lung, and the mediastinal lymph nodes were also enlarged. Whole-body positron emission tomography-computed tomography (PET-CT) showed hypermetabolic lymph nodes in the mediastinum (4R group) and metastatic lymph nodes with an unknown primary focus, although the metabolism of the left lung was not high (**Figure 1D**). Subsequent endobronchial ultrasound-guided fine-needle aspiration biopsy for the mediastinal (group 4R) metabolic lymph nodes showed undifferentiated small cell cancer (**Figure 1E**). Pathological immunohistochemistry revealed the following: CK-pan (±), TTF-1 (+), Syn (±), LCA (+), and CD56 (±).

### 2.2 Case 2

**Figure 3:**
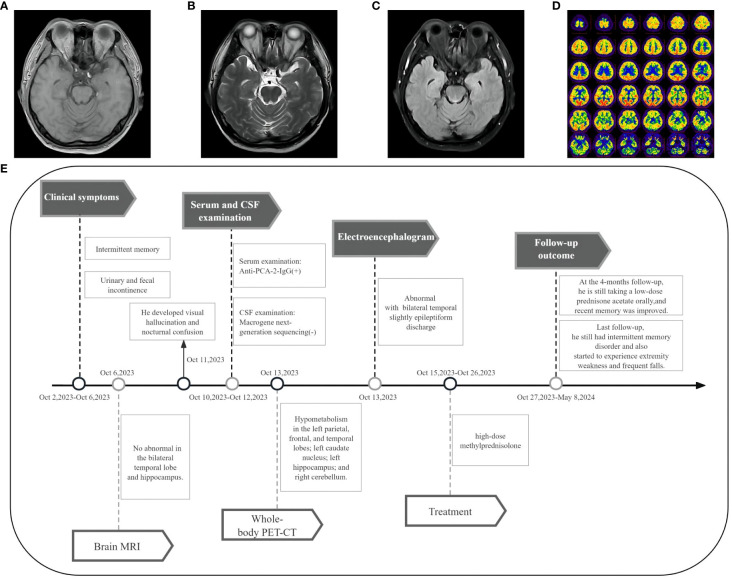
Imaging and clinical course of Case 2. **(A-C)** Brain MRI showed no abnormal signals in the bilateral temporal lobe and hippocampus. **(D)** PET-CT showed hypometabolism in the left parietal lobe, frontal lobe, temporal lobe, left caudate nucleus, left hippocampus, and right cerebellum. **(E)** Timeline of the clinical course in Case 2.

A 45-year-old male patient, a smoker for 25 years, presented with intermittent memory confusion lasting 6–7 days. This manifested as forgetting his mobile phone password and not recognizing colleagues. He also experienced two episodes of urinary and fecal incontinence but maintained clear consciousness and normal responsiveness. The patient had recently lost approximately 5 kg in weight. Four days after admission, he developed visual hallucinations and nocturnal confusion. He did not experience dizziness, walking instability, or limb convulsions throughout the course of his illness. Neurological examination showed slightly poor orientation, significantly decreased recent memory, and slightly decreased long-term memory; no other abnormalities were noted. The brain MRI of the bilateral temporal lobe and hippocampus was normal (**Figures 3A–C**). However, a 24-hour electroencephalogram displayed slight epileptiform discharges in the bilateral temporal lobes.

CSF examination by lumbar puncture elevated levels of protein (0.9 g/L [reference range: 0.15–0.45 g/L]) and immunoglobulin G (80.33 mg/L [reference range: 0–34 mg/L]). Macrogene next-generation sequencing of the CSF sample yielded negative findings, but cytokeratin 19 levels were elevated at 6.33 ng/mL (reference range: <5.0 ng/mL). A lung CT scan revealed inflammatory nodules (~0.4–0.7 cm) in the right lobes of the lung. The tissue-based assay was positive for serum paraneoplastic antibody, and the cell-based assay was positive for anti-PCA-2 (MAP1B) (titer 1:30, **Figures 2A, B, E**). Meanwhile, anti-NMDAR, AMPA1, AMPA2, LGI1, CASPR2, GlyR1, GABA_A_R, GABA_B_R, IgLON5, DPPX, DRD2, GAD65, mGluR1, mGluR5, neurexin-3α, ganalionic AchR, KLHL11, GluK2, AK5, AGO, CaVα2δ, AQP4, MOG, and GFAP were tested negative. Granular-type (1:3200) antinuclear antibodies were also found, but not other types of antinuclear antibodies. The Rheumatology and Immunology Department indicated that the presence of a single antinuclear antibody particle type was a secondary change. To further screen for the primary cause, the patient underwent whole-body PET-CT imaging, which revealed hypometabolism in the left parietal, frontal, and temporal lobes; left caudate nucleus; left hippocampus; and right cerebellum (**Figure 3D**). There was low metabolism in the nodules in the upper lobe of the right lung and high density in the gallbladder cavity, either from the contrast agent or bile stasis. Other considerations included gallbladder neck duct stones and double renal pelvis and right ureteral calculi with secondary right renal pelvis and right middle and upper ureteral dilated effusions. No tumor was found during PET-CT imaging.

## 3 Timeline

The timeline of case 1 (**Figure 1F**).

The timeline of case 2 (**Figure 3E**).

## 4 Diagnostic assessment, treatment, and follow-up outcomes

### 4.1 Case 1

Patient 1 presented with subacute onset and clinical manifestations of cerebellar damage such as dizziness and an unsteady gait. Laboratory tests revealed leukocytosis (> 5×10^6/L) in CSF, and the cell-based assay revealed positivity for anti-PCA-2 (MAP1B) in serum and CSF. Undifferentiated small cell cancer found in mediastinal (group 4R) metabolic lymph nodes is also the most common tumor type associated with anti-PCA-2. The patient was finally diagnosed with PCA-2-associated autoimmune cerebellitis and undifferentiated small cell carcinoma with mediastinal lymph node metastasis of unclear primary focus. He was subsequently transferred to the Department of Oncology, diagnosed with lung cancer (small-cell TXN2M0 IIIA stage), and was administered chemotherapy with 3 cycles of etoposide (200 mg on day 1 and 150 mg on days 2–3) and carboplatin (500 mg on day 1). Mediastinal (group 4R) metabolic lymph node radiation was started, and 60 Gy was delivered in 30 fractions of 2 Gy each over 42 elapsed days after cycle 3 of chemotherapy. At the 6-month follow-up, his dizziness had improved, but gait instability symptoms worsened, and he could not walk unassisted. At the last follow-up, the patient had stable disease, but he developed limb numbness. Thus, he was recommended for repeat tests for paraneoplastic syndrome-related antibodies to clarify the current titer change of PCA-2 and whether it was combined with other types of antibodies. Additional immunotherapy was also recommended to improve gait instability. However, immunotherapy was refused owing to concerns about the possibility of increasing the primary tumor focus with steroids and other immunotherapies.

### 4.2 Case 2

Patient 2 had limbic system symptoms such as decreased recent memory and abnormal mental behavior. The CSF examination showed elevated levels of protein and immunoglobulin G. Whole-body PET-CT imaging revealed hypometabolism in the left parietal, frontal, and temporal lobes, left caudate nucleus, left hippocampus, and right cerebellum. The electroencephalogram displayed slight epileptiform discharges in the bilateral temporal lobes. Moreover, the cell-based assay was positive for anti-PCA-2 (MAP1B). Finally, the patient was diagnosed with PCA-2-associated limbic encephalitis and treated with 12 days of intravenous methylprednisolone pulse-therapy (1 g on days 1–3, 0.5 g on days 4–6, 0.24 g on days 7–9, and 0.12 g on days 10–12), followed by oral prednisone acetate (60 mg/day, reduced by 5 mg every two weeks). To date, 8 months after initial presentation, the patient is still on oral prednisone acetate therapy (5 mg/day). At the 4-month follow-up, his recent memory had improved, but he still had intermittent memory disorder. At the last follow-up, the patient also started to experience extremity weakness and frequent falls. He also experienced a significant weight loss of approximately 15 kg. Repeated paraneoplastic antibody tests in the serum and CSF and PET-CT were recommended to screen for tumors. However, the patient refused owing to economic reasons and preferred to continue the oral low-dose prednisone acetate treatment.

## 5 Discussion

PCAs, including PCA-1 (Yo), PCA-2 (MAP1B), and PCA-Tr (DNER), are high-risk antibodies for PNS and can cause autoimmune cerebellar ataxia. Although the symptoms of autoimmune cerebellar ataxia caused by anti-PCAs antibodies may be similar, there are differences in several aspects such as sex, tumor occurrence, merging of other antibodies, and treatment effects (4, 5). Moreover, because MAP1B is widely distributed in the central and peripheral nervous systems, there are more clinical manifestations in PCA-2-related nervous system diseases than in other PCA-related diseases. In a recent analysis of PCA-2-related diseases using data from the Mayo Clinic, 61% of the 95 patients with clinically verifiable information had subacute signs and symptoms. The neurological manifestations varied, ranging from a single symptom to a combination of several symptoms. Approximately 47% of the patients had multiple clinical manifestations, and the main clinical manifestations were peripheral neuropathy (53%), somatesthesia disorder (45%), cerebellar ataxia (32%), encephalopathy/cognitive decline (27%), gastrointestinal motility disorder (8%), borderline encephalitis (7%), and Parkinson’s/dystonia/chorea (5%) (2). Although both patients in this study had PCA-2-related neurological diseases, the onset forms were completely different; Patient 1 developed the disease in the form of unstable gait, dizziness, and poetic-like language, whereas Patient 2 presented with intermittent memory disorder and also developed visual hallucinations and nocturnal confusion during the disease course, reflecting the varied clinical phenotypes of PCA-2-related diseases.

PCA-2-associated limbic encephalitis accounts for only 7% of all PCA-2-related neurological diseases. Currently, only 10 cases of PCA-2-associated limbic encephalitis have been verified (**Supplementary Table 1**) (2, 6), but almost all of these cases were combined with other antibodies. In our study, only a single PCA-2 was found in Patient 2, suggesting that PCA-2 could be the responsible antibody for limbic encephalitis. Therefore, when diagnosing and treating limbic encephalitis, the possibility of PCA-2 should be considered in addition to anti-NMDAR, anti-LGI1 and anti-GABAR, which are common causes of marginal lobe encephalitis. The main differences between PCA-2 related limbic encephalitis and common limbic encephalitis are in clinical manifestations, tumor occurrence, and treatment effects. The clinical manifestations of anti-NMDAR encephalitis usually include prodromal symptoms such as fever and headache, followed by obvious neurological and psychiatric symptoms such as blurred consciousness, epileptic seizures, and autonomic instability, which usually require hospitalization in the intensive care unit (7). Approximately 38% of cases are associated with tumors, which are predominant among middle-aged and young women, are mainly ovarian teratomas (4), and are sensitive to immunotherapy. Anti-GABA_B_R limbic encephalitis is more common in elderly males with smoking habits, and its clinical manifestations often include behavioral changes and refractory epileptic seizures. More than 50% of cases are related to tumors, with the most common being small cell lung cancer (4). The characteristic clinical manifestations of anti-LGI1 limbic encephalitis include the subacute onset of memory decline, epileptic seizures, and/or facial arm muscle tone disorders, with a less than 10% probability of concomitant tumors (4). Its recurrence rate of anti-LGI1 limbic encephalitis is high and immunotherapy is effective at the beginning of the disease (8).

PCA-2 is a high-risk antibody for tumor development, with the tumor probability being as high as 79%. Tumors can appear simultaneously with antibody positivity or before or after antibody positivity. In a study by Gadoth et al., tumors were found after and before antibody positivity in 43% and 17% of patients, respectively; 20% of the patients had no tumor after screening. Tumor and neurological symptoms occurred simultaneously in 9% of the patients (2). However, there are currently no data on the median time to tumor detection after the onset of neurological symptoms because the updated PNS criteria indicate that patients with high-risk antibodies need to undergo repeat tumor screening every 4–6 months for 2 years. Knowing the median time to detect tumors after the onset of neurological symptoms will be helpful in developing appropriate guidelines for the regular screening for tumors in PCA-2-positive patients. Moreover, PCA-2 is more likely to be combined with other antibodies; approximately 67% of patients with PCA-2 have other antibodies (2). The most common co-existing antibodies are CRMP5 (26%), P/Q-VGCC (21%), GAD65 (15%), ANNA-1 (13%), VGKC (Kv1)-complex (7%), nicotinic ganglionic (α3) AChR (6%), nicotinic muscle AChR (3%), GABA-B receptor (3%), amphiphysin (2%), N-VGCC (2%), AMPA receptor (2%), and AGNA-1 (SOX-1) (2%) (2). Various clinical manifestations associated with PCA-2 may also be related to these co-existing antibodies, but the antibodies responsible for each clinical manifestation are yet to be defined.

In the study by Jitprapaikulsan et al., all patients with PCA-2 co-existing with CRMP5 or ANNA1 antibodies had cancer, indicating the importance of antibody clusters in predicting underlying cancers (9). Although PCA-2 often co-exists with other onconeural autoantibodies, the lung cancer detection rate is similar between PCA-2 patients with and without co-existing onconeural autoantibodies (66% and 63%, respectively) (2). In addition, PCA-2 is not only associated with tumors but also with neurological diseases after the use of immune checkpoint inhibitors (ICIs) in tumor therapy. To date, five patients with PCA-2-related neurological diseases have been reported (**Supplementary Table 2**) (6, 10–13). One patient developed PCA-2-related neurological disease after treatment with a PD-1 inhibitor. In this patient, it is unclear whether the presence of PCA-2 was a pathological change induced by the tumor itself or by ICIs. PCA-2-related adverse events after the use of ICIs require further research.

The current two patients showed different MRI and PET findings. Patient 1 initially did not show abnormal signals or atrophy in the cerebellum even 6 months after disease onset. However, cerebellar atrophy might be found on brain MRI in the later stage of disease progression, suggesting the need for long-term imaging follow-up. Patient 2 did not show abnormal signals in the bilateral temporal lobe and cerebellum by brain MRI examination 4 days after disease onset, but his whole-body PET-CT showed hypometabolism in the left parietal lobe, frontal lobe, temporal lobe, left caudate nucleus, left hippocampus, and right cerebellar brain after 10 days. Moreover, Patient 2 did not show symptoms of cerebellar ataxia, but PET-CT showed cerebellar hypometabolism. We believe that this may be a subclinical molecular metabolic lesion itself or a cerebrum lesion that led to cerebellar cross disconnection.

Vernino et al. reported a case of limbic encephalitis in which the brain MRI was normal at the beginning, but the subsequent brain MRI showed abnormalities in the left medial temporal lobe (1). This supports the idea that normal brain MRI findings should not be used as the basis for ruling out limbic encephalitis. Lesions can be detected on PET-CT during the early stages or on the follow-up brain MRI. Although abnormal signals in the bilateral temporal lobe on brain MRI are one of the diagnostic criteria for autoimmune encephalitis (AE), some patients with AE show normal findings at disease onset. In one study, MRI changes were observed in only 40% of cases, but PET-CT findings were abnormal in 82%. In addition, PET-CT showed more abnormalities than initial electroencephalogram, MRI, and CSF tests in patients with AE, and the most common abnormality was hypometabolism in brain regions. PET-CT imaging may be more sensitive than MRI in showing increased fluorodeoxyglucose metabolism in normal-appearing medial temporal lobes (14).

Antibody-related AE shows specific metabolic patterns. In those related to neuronal surface antibodies (e.g., anti-NMDAR encephalitis), the metabolism of the frontal parietal and medial temporal lobes is increased, whereas that of the occipital lobe is decreased. In anti-LGI1 encephalitis, abnormal metabolism occurs in different brain regions, including the medial temporal, hippocampus, and cerebellum. Patients with facial arm dystonia show basal ganglia abnormalities. In AEs related to neuronal intracellular antibodies, hypometabolism is observed in the parietal, frontal, occipital, temporal, and hippocampus lobes in patients with anti-GAD65 and anti-Ma2 encephalitis. Patients with anti-Hu encephalitis show both hypometabolism and hypermetabolism in the medial temporal lobe and hypometabolism in cortical regions (e.g., frontal, occipital, and parietal lobes) (15). Therefore, the position of the antigen bound by antibodies inside and outside the cell influences the metabolism of the lesion site on PET-CT. Furthermore, as this position relates to different metabolic changes on PET-CT, PET-CT can be used for the early differentiation of limbic encephalitis caused by different autoimmune antibodies, particularly those caused by neuronal intracellular and extracellular antigens. However, there are limited reports on the PET-CT imaging findings of PCA-2-associated limbic encephalitis, emphasizing the need for further studies.

Traditional immunotherapy such as steroids, immunoglobulin, and plasmapheresis may only moderately improve symptoms, and approximately 50% of patients have a poor response. The general effect may be attributed to antibody targeting of neuronal antigens. The reason why traditional immunotherapy has a general effect may be that the target antigen of this kind of antibody is in the neuron, which is not easily accessed by the antibody; thus, this type of antibody does not directly produce pathological effects but attacks the neuronal antigen through the immune response mediated by T cells. In patients with PNS, CD3+ and CD8+ T-lymphocytes predominantly infiltrate the brain or dorsal root ganglia, supporting cytotoxic T-cell-mediated neuronal loss rather than antibody-mediated dysfunction (16, 17). However, antibodies against cell surface antigens can bind to cell surface proteins on living neurons, and postmortem tissue examination shows that anti-IgG deposition around neurons does not lead to substantial cell death (17). Thus, diseases associated with antibodies to cell surface antigens have a better response to immunotherapy, whereas those associated with intracellular neuronal antibodies have a poor response. PCA-2-associated neurological diseases have various phenotypes, but those with a poor response to traditional immunotherapy have not been identified. One prospective study showed that twice as many patients treated with a combination of plasmapheresis and cyclophosphamide had improvements in disability as those treated with plasmapheresis alone. Therefore, for patients with a poor response to first-line immunotherapy, more aggressive immunosuppression with early initiation of second-line agents such as cyclophosphamide should be considered (18).

The treatment strategies differ for patients with positive PCA-2 with and without concurrent tumors. In Patient 1, treatment was focused on the primary lesion, and thus, there was no attempt at immunotherapy. However, despite chemotherapy and radiotherapy, the patient’s gait instability worsened. This indicates that T-cell-mediated Purkinje cell destruction is irreversible. Moreover, Patient 2 had no tumor and was treated with intravenous methylprednisolone immunotherapy, and the symptoms of memory confusion and senseless talking were significantly improved. This indicates that some patients still respond to first-line immunotherapy. On follow-up, Patient 1 developed limb numbness, and Patient 2 developed limb weakness and consequently experienced frequent falls. This newly developed neurological symptom may be related to PCA-2 itself or in combination with other antibodies. PCA-2 is often combined with CRMP5 and/or ANNA1 antibodies, and they can cause sensory or motor neuropathy. However, peripheral nerve examination was not performed in both patients; thus, it was impossible to determine the existence of peripheral neuropathy. Moreover, there were no tests for CRMP5, ANNA1, and other antibodies; as such, it was unclear whether other antibodies, which may have caused new symptoms, were present. Prognostic differences among PCA-2 phenotypes have not been reported, but patients with peripheral neuropathy with co-existing PCA-2 and ANNA1 antibodies and/or CRMP5 antibodies have a lower survival rate (9).

In conclusion, we report two cases of PCA-2-associated encephalitis with completely different onset forms, clinical manifestations, imaging findings, tumorigenesis, and therapeutic responses. The limitation of this work is that neither patient was retested for antibodies; therefore, it was unclear whether the new symptoms were related to changes in anti-PCA-2 antibody titer or the combination of other antibodies. In future research, we should focus on the potential correlation between antibody titer and symptom severity. Finally, given the high risk of relapse and the poor effect of traditional immunotherapy on PCA-2-related neurological diseases, future studies should pay more attention to their pathogenesis to establish more effective treatment methods for controlling symptoms and improving prognosis.

## Data availability statement

The original contributions presented in the study are included in the article/**Supplementary Material**. Further inquiries can be directed to the corresponding author.

## Ethics statement

Written informed consent was obtained from the individual(s) for the publication of any potentially identifiable images or data included in this article.

## Author contributions

XL: Conceptualization, Data curation, Software, Writing – original draft, Writing – review & editing. YL: Conceptualization, Data curation, Software, Writing – original draft, Writing – review & editing. DM: Formal analysis, Resources, Writing – review & editing. JB: Formal analysis, Resources, Writing – review & editing. PS: Formal analysis, Writing – review & editing, Resources. XW: Writing – review & editing, Formal analysis, Resources. LC: Funding acquisition, Investigation, Project administration, Supervision, Writing – review & editing.
