# Supplementary material for: Case report: PCA-2-associated encephalitis with different clinical phenotypes: a two-case series and literature review
Source: Front Immunol. 2024 Jul 12;15:1431585. doi: 10.3389/fimmu.2024.1431585 (PMC11272519; doi:10.3389/fimmu.2024.1431585)
Supplement: Supplementary file 1 [file Table_1.docx]

Supplementary Material

PCA-2–associated encephalitis with different clinical phenotypes: A Two-Case Series and literature review

**Xiaona Li, Yue Lang, Di Ma, Jing Bai, Pingping Shen, Xinyu Wang, Li Cui^*^**

*** Correspondence:** Li Cui, lcui@jlu.edu.cn

**Supplementary Table 1. Summary of characteristics of patients with PCA-2–related limbic encephalitis in the literature**

| Pt No | Sex/Age | Clinical syndrome | Additional antibodies | Cancer/time of diagnosis | MRI brain | MRI spine | CSF WCC*/Pro/OCBs/ IgG I | Treatment /response |
| --- | --- | --- | --- | --- | --- | --- | --- | --- |
| 1 | F/60 | Limbic encephalitis, seizures and myelitis | CCPQ, Alpha3, GAD 65 | SCLC/follow | HC lesion | NA | NA | NA |
| 2 | M/80 | Limbic encephalitis, motor weakness | CRMP-5, STR, GAD-65, VGKC | NA | NA | NA | I/I/NA/NA | NA |
| 3 | M/73 | Limbic encephalitis |  | SCLC/prior | NA | NA | NA | NA |
| 4 | M/75 | Limbic encephalitis | ANNA-1, CRMP-5 | NSCLC/follow | Temporal and parietal lobes signal abnormalities | NA | 1/I/1/I | NA |
| 5 | F/66 | Limbic encephalitis, ataxia, sensory neuropathy, chorea, decreased vision | CRMP-5, Amphi | SCLC/follow | T2 hyperintensities in the basal ganglia | NA | I/I/NA/NA | Chemo,steroids/stable |
| 6 | F/79 | Limbic encephalitis | ANNA-1 | No | Temporal hyperintensities and hippocampal atrophy | NA | 1/I/NA/NA | No |
| 7 | M/73 | Limbic encephalitis, gait unsteadiness | ANNA-1 | SCLC/follow | atrophy and scattered T2 white matter hyperintensities | mild contrast prominence in the inferior thoracic spine | I/I/NA/NA | Chemo, steroids/Yes |
| 8 | M/70 | Limbic encephalitis | No | SCLC/follow | NA | NA | NA | NA |
| 9 | F/67 | Limbic encephalitis, seizures | GABA-B, AMPA, CCN | Lung cancer/NA | NA | NA | NA | Chemo/NA |
| 10 | M/17 | Limbic encephalitis | SOX-1 | Hodgkin lymphoma/prior | hyperintense lesions with contrast enhancement in the medial temporal lobe and limbic system | NA | NA/I/NA/NA | steroids, IG, Chemo,Rad, CTX, PLX/relapsed after reducing therapy |

CCPQ = Calcium Channel type P/Q,Alpha-3 = Nicotinic ganglionic acetylcholine receptor,STR = striational antibodies,AGNA-1 = Anti-glial/neuronal nuclear antibodies (also known as Sox1), Amphi = amphiphysin, CCN = Calcium Channel type N,Prior = cancer diagnosed prior to neurological symptoms,Follow = cancer diagnosed following neurological symptoms, HC = hippocampal, NA = Not applicable/not available, NSCLC=Non-small cell lung cancer,SCLC=small cell lung cancer,I = increased/elevated, Pro=protein,WCC= white cells, OCBs=CSF - exclusive oligoclonal bands,IgG I=IgG index,Chemo = chemotherapy, PLX = plasma exchange,Rad = radiation,CTX = cytoxane,IG=immunoglobulin

**Supplementary Table 2. Summary of characteristics of patients with PCA-2–associated neurological diseases in the literature**

| Pt No | Sex/age (years) | Clinical syndrome | Additional antibody/time of diagnosis | Cancer/time of diagnosis | MRI brain/spine | CSF WBC*/Pro/OCBs/ IgG I | Treatment | Prognosis |
| --- | --- | --- | --- | --- | --- | --- | --- | --- |
| 1 | M/57 | Small fiber neuropathy, chorea | CRMP-5, follow | SCLC, follow | Asymmetrically increased T2-FLAIR signal involving the head of the right caudate nucleus and putamen/NA | I/NL/I/I | CDDP, etoposide, Rad, MP | The PCA-2 titer is decreased, but the patient still has neurological deficits after 1 year |
| 2 | M/17 | Limbic encephalitis | SOX-1, prior | Hodgkin lymphoma/prior | Hyperintense lesions with contrast enhancement in the medial temporal lobe and limbic system/NA | I/NA/NA/NA | Steroids, IG, chemo, Rad, CTX, PLX | After dose reduction or discontinuation of immunosuppressive therapy, the patient develops relapse, and is still on oral prednisone 5 years later. The patient is able to walk with walking aid and orthotics |
| 3 | F/50 | Subacute cerebellar syndrome | None | Renal cell carcinoma with lung metastases/prior | Hyperintensity of the cerebellum/NA | NL/NL/NA/NA | DXM | Significant improvement in dizziness and instability |
| 4 | F/57 | Optic neuropathy, vitritis, and peripheral neuropathy | None | SCLC/at | Scattered periventricular, deep white matter changes on FLAIR sequences/NL | NA | Carboplatin, etoposide, rad | Patient’s vision and neuropathy remained stable |
| 5 | M/70 | Chorea, dystonia syndrome | None | Suspected malignancy in the left mediastinal area/NA | Bilateral caudate atrophy along with global parenchymal loss/NL | NA | PLX | No improvement |

Prior: cancer diagnosed prior to neurological symptoms/other antibodies detected prior to PCA-2, follow: cancer diagnosed following neurological symptoms/other antibodies detected following PCA-2, at: cancer diagnosed at presentation of neurological symptoms/other antibodies detected with PCA-2, NA: not applicable/not available, NL: normal, NSCLC: non-small cell lung cancer, SCLC: small cell lung cancer, Pro: protein, WBC: white blood cells, OCBs: CSF - exclusive oligoclonal bands, IgG I: IgG index, I: increased/elevated, Chemo: chemotherapy,DXM=dexamethasone, PLX: plasma exchange, Rad: radiation, CTX: cytoxan, IG: immunoglobulin, CDDP: cisplatin, MP: methylprednisolone
